# Overcoming urea and nitrate inhibition of biological nitrogen fixation in *Azotobacter vinelandii* versus natural tolerance in *Gluconacetobacter diazotrophicus*

**DOI:** 10.1007/s11274-026-05209-0

**Published:** 2026-09-01

**Authors:** Donnen Yong, Brett M. Barney

**Affiliations:** 1 Department of Bioproducts and Biosystems Engineering, University of Minnesota, 1390 Eckles Avenue, Saint Paul, MN 55108-6130 USA; 2 Biotechnology Institute, University of Minnesota, Saint Paul, MN 55108 USA

**Keywords:** *Azotobacter vinelandii*, Urea, Nitrate, Nitrogen fixation

## Abstract

Nitrogen is often a limiting nutrient for agricultural crops. Most microbes also require an external fixed-nitrogen source for optimal growth. Even for microbes capable of biological nitrogen fixation (BNF), it is generally the case that they will regulate their metabolism to prioritize assimilation of fixed-nitrogen. Microbes employ various strategies and pathways to take advantage of available fixed-nitrogen compounds found in their natural environments. *Azotobacter vinelandii* is a model microbe for the study of BNF. Due to the high energetic cost of the process, BNF in *A. vinelandii* is repressed in the presence of ammonium, urea and nitrate. Prior studies indicated that strong inhibition of nitrogen fixation by urea and nitrate in *A. vinelandii* is actually the result of intracellular conversions of these metabolites into ammonium. In this study, we demonstrate a strategy to eliminate BNF inhibition by both urea and nitrate at concentrations ranging as high as 15 mM in a strain lacking the genes for urease and nitrate reductase, resulting in continued nitrogenase activity in the presence of these common fertilizer inputs. In contrast to the properties of *A. vinelandii*, the diazotroph *Gluconacetobacter diazotrophicus* naturally lacks these pathways, prompting the question of whether urea or nitrate inhibit BNF or support growth in *G. diazotrophicus*. To probe this observation, we developed experiments to demonstrate that while the presence of urea and nitrate delay the initial growth rate in *G. diazotrophicus*, nitrogenase activity and ammonium accumulation occurs at a similar rate in the presence of these metabolites. These results indicate that biological nitrogen fixation in *G. diazotrophicus* is somewhat insensitive to these nitrogen sources. This illustrates that alternative pathways in diazotrophic strains should be carefully considered in any efforts to optimize extracellular nitrogen production for biofertilizer applications and strain optimization, and that additional design strategies can be effective to assure that diazotrophs continue to fix nitrogen in the presence of specific nitrogen compounds common to industrial fertilizers.

## Introduction

Our growing global population is predicted to be a central driver of increased food requirements over the coming decades, with demand expected to grow substantially through 2050 based on certain projections (Dijk et al. 2021). This ever-increasing requirement for food will necessitate additional crop production, which can be achieved through increased or improved land use, better utilization of water resources, and lowered agricultural inputs such as fertilizers (Tilman et al. 2011). Nitrogen-based chemical fertilizers are central to global crop production, exceeding the combined use of phosphorus and potassium fertilizers (Nguyen et al. 2024), with urea accounting for the largest percentage of nitrogen-based fertilizer (Heffer and Prud’homme 2016). Urea is produced on industrial scales through the Bosch-Meiser process (Mao et al. 2024) and nitrate is produced through the Ostwald process (Michalski et al. 2015). Both of these processes begin with ammonia, which is first produced by the Haber-Bosch process, an energy intensive process that accounts for ~ 1–2% of global energy consumption and CO_2_ emissions annually (Smil 2001; Smith 2002; Smith et al. 2020; Zhou et al. 2017). Recent alternatives to the Haber-Bosch process seem promising, but remain unadopted by industry (Canfield et al. 2010; Mao et al. 2024; Smil 1999; Smith et al. 2020). In addition, synthetic fertilizers contribute to environmental degradation through nutrient runoff, groundwater contamination, and greenhouse gas emissions such as nitrous oxide, whilst depleting soil health and microbial diversity over time (Bijay and Craswell 2021; Geisseler and Scow 2014; Shcherbak et al. 2014). Biofertilizer strains are microbes that could serve as a sustainable alternative to synthetic fertilizers. In the case of nitrogen fertilization, diazotrophic microorganisms capable of biological nitrogen fixation (BNF), convert atmospheric nitrogen into ammonia through the enzyme nitrogenase, which is then made available to associated plants (Ladha et al. 2022).

Diazotrophic microbes are found in many ecosystems, but are perhaps best recognized for the specific associations they form with agriculturally relevant plants, including the model symbiotic association formed between rhizobia and root nodules in legumes (Rhijn and Vanderleyden 1995). There are many additional diazotrophs associated with the rhizosphere surrounding plant roots that do not require nodules to form close associations with the plant (Islam et al. 2009; Rosenblueth et al. 2018; Ryu et al. 2020). These include microbes that are associated with the surface of the plant (epiphytes) and those that reside inside the plant roots (endophytes). Many of these microbes are of interest for potential applications to increase soil nitrogen through BNF, including *Azospirillum* spp., *Azoarcus* spp., *Klebsiella* spp., *Herbaspirillum* spp., *Gluconacetobacter diazotrophicus*, and a variety of others (Bertalan et al. 2009; Faoro et al. 2017; Kaneko et al. 2010, Krause et al. 2006; Pavlova et al. 2017; Rosconi et al. 2016). Additional free-living diazotrophs include the model microbe *Azotobacter vinelandii*, which has been studied in laboratories around the world for more than a century as a model system for the study of BNF (Barney 2024; Jensen 1954; Noar and Bruno-Bárcena 2018).

Prior studies with the model diazotrophs *Azotobacter vinelandii*, and *Gluconacetobacter diazotrophicus* have demonstrated the ability to increase the extracellular production of ammonium and urea from these microbes (Barney and Dietz 2024, 2025; Barney et al. 2015; Dietz et al. 2024). For ammonium overproduction, many strategies involve the deregulation of biological nitrogen fixation, often by disrupting either the global regulatory modulators or by making ammonium assimilation less efficient (Bali et al. 1992, Colnaghi et al. 1997; Thomas et al. 1990). In the absence of dinitrogen gas, the nitrogenase enzyme will reduce protons into hydrogen gas (Burgess and Lowe 1996; Igarashi et al. 2005). As part of a prior study to optimize aerobic hydrogen production through nitrogenase in *A. vinelandii*, our laboratory tested BNF inhibition by various forms of fixed nitrogen, and demonstrated that urea and nitrate do not inhibit BNF when pathways that convert these to ammonium are absent (Knutson et al. 2018). This observation could impact efforts to develop biofertilizer applications that are administered along with industrial fertilizers.

The goal of this study was to test the inhibitory effects of specific nitrogen compounds on BNF, particularly those commonly found in industrial fertilizers, and evaluate the potential to eliminate this inhibition through the disruption or removal of multiple pathways. It is well established that ammonium is a strong inhibitor of BNF in *A. vinelandii* (Klugkist and Haaker 1984), but much less is known about urea and nitrate (Gusso et al. 2008; Sorger 1969). Understanding the impacts of plant-available nitrogen compounds found in many soils (Farzadfar et al. 2021) will be essential to future strategies to optimize BNF and harness diazotrophs as an approach to lower our dependence on the Haber Bosch process (Erisman et al. 2008; Smith et al. 2020). This study demonstrates a simple approach to improve performance of biofertilizer strains in the presence of nitrate and urea, which could be applied to a much broader range of diazotrophs in biofertilizer applications, and identifies *G. diazotrophicus* as a microbe that already employs such a strategy.

## Materials and methods

*Azotobacter vinelandii* DJ (ATCC BAA-1303) was obtained from Dennis Dean. *A. vinelandii* strains were grown aerobically at 27 °C on agar plates and liquid Burk’s (B) medium containing (per liter): 20 g Sucrose, 800 mg K_2_HPO_4_, 200 mg KH_2_PO_4_, 200 mg MgSO_4_·7H_2_O, 90 mg CaCl_2_·2H_2_O, 5 mg FeSO_4_·7H_2_O, and 0.25 mg Na_2_MoO_4_·2H_2_O. To test for nitrogenase repression, B medium was supplemented with urea, ammonium sulfate, sodium nitrate or sodium nitrite. *Gluconacetobacter diazotrophicus* PA1 5 (ATCC 49037) was obtained from Cedric Owens. *G. diazotrophicus* GABB034 (Δ*amtB1* Δ*amtB2*) was constructed as previously described (Dietz et al. 2024). *G. diazotrophicus* strains were grown and maintained on GADN medium (Schwister et al. 2022). For diazotrophic growth, GABB034 was grown on B medium supplemented with 35 mM sodium citrate and 1.25 mM asparagine and adjusted to pH 6.8. To test for the ability to utilize urea or nitrate, wild-type *G. diazotrophicus* and GABB034 were both grown on the same media described above, and on media containing 10 mM urea in place of asparagine or 20 mM sodium nitrate in place of asparagine. Plasmids used to build new strains were constructed and maintained within *Escherichia coli* JM109 obtained from New England Biolabs (Ipswich, MA).

### Strain construction

*A. vinelandii* strain AZBB883 was constructed from strain AZBB120 (Δ*ureABC* and Δ*amtB*), which lacks a functional urease and ammonium transporter, and which was previously described (Table 1 and (Eberhart et al. 2016)). First, AZBB120 was transformed with pPCRURE92 (Table 2) to replace the *nasAB* genes encoding nitrate and nitrite reductase with a tetracycline selective marker and *lacZ* gene for identification using X-Gal, yielding strain AZBB865. Next, AZBB865 was transformed with pPCRURE101 by single homologous recombination to add *sacB* and a kanamycin selective marker to prime the strain for counter-selection, yielding strain AZBB871. Finally, strain AZBB871 was cultured in glucose-substituted B medium without antibiotics overnight to allow the strain to recombine. The strain was transferred to B medium with sucrose to induce conditional toxicity for two transfers, and then cells were diluted and plated onto B plates with X-Gal to identify strains lacking the *nasAB* genes, resulting in strain AZBB883 (Δ*nasAB*, Δ*ureABC* and Δ*amtB*). The final strain was confirmed by performing colony PCR with primer set BBP1397 and BBP2394 (Table 3). A general description of this approach has been described (Dietz et al. 2024).

**Table 1:** Mutant strains constructed and/or used in this study

*A. vinelandii* Strain	Plasmid utilized	Genetic features	Parent strain/reference
*Azotobacter vinelandii* DJ	None	Wild-type	
AZBB120		Δ*ureABC* and Δ*amtB*	(Eberhart et al. 2016)
AZBB865	pPCRURE92	*nasAB*::tet^R^-*lacZ*, Δ*ureABC* and Δ*amtB*	AZBB120
AZBB871	pPCRURE101	*nasAB*::tet^R^-*lacZ* with single homologous recombination of pPCRURE101, Δ*ureABC* and Δ*amtB*	AZBB865
AZBB883		Δ*nasAB*, Δ*ureABC* and Δ*amtB*	AZBB871
*Gluconacetobacter diazotrophicus*	None	Wild-type	
GABB034		Δ*amtB1* and Δ*amtB2*	(Dietz et al. 2024)

**Table 2:** Key plasmids and relevant derivatives of these plasmids used for the construction of Azotobacter vinelandii manipulated strains

Plasmid^a^	Relevant genes cloned or plasmid manipulation	Parent Vector(s)	Reference and/or source
pBB053	pUC19 derivative.		(Lenneman et al. 2013)
pLACZF19	Plasmid containing *lacZ* and tetracycline cassette.		(Dietz et al. 2024)
pPCRNRK10	Cloned *nasAB* genes and flanking regions into pBB053.	pBB053	This Study
pPCRNRK11-2	Removed *nasAB* genes from pPCRNRK10, leaving flanking regions behind.	pPCRNRK10	This Study
pPCRSACB31	Restriction site optimized plasmid containing *sacB* and kanamycin resistance cassette.	pPCRSACB28	(Dietz et al. 2024)
pPCRURE92	Inserted tetracycline and *lacZ* gene cassette from pLACZF19 between flanking regions of pPCRNRK11-2 for removal of *nasAB* genes.	pPCRNRK11-2, pLAZCF19	This Study
pPCRURE101	Moved *sacB* and kanamycin selection cassette from pPCRSACB31 into pPCRNRK11-2 for counter-selection construction.	pPCRNRK11-2, pPCRSACB31	This Study

^a^ Sequences for all plasmids listed are available upon request

**Table 3:** Primers used for the construction of *Azotobacter vinelandii* manipulated strains

Primer	Sequence (all in 5’ to 3’ direction)	Plasmid construct
BBP1237	GACTAAGCTT CTCGACTTCG GCCTGGCCTA CTG	pPCRNRK10
BBP2376	NNNTCTAGAC AGTTCAGGCT GCTGTCGTCC TGGGTGTG	pPCRNRK10
BBP2377	NNNAGATCTT CTCCGTTACG GAAAGCGG	pPCRNRK11-2
BBP2378	NNNAGATCTG AATCCGGGAC GGAAGAGCGC ACAAC	pPCRNRK11-2
BBP1397	CTGTTGCTGG AGGAATGGTT CCTGCGTC	
BBP2394	CGTGATGTTC TGGTCAACGA GTATGGC	

### Acetylene reduction assays

Whole cell acetylene reduction assays were conducted with *A. vinelandii* to measure nitrogenase activity. Cells were transferred from a two-day old agar plate into 3 mL of liquid medium supplemented with or without ammonium, urea, or nitrate in 20 mL glass serum vials with a target OD_600_ of 1.0. The vials were capped and sealed with rubber stoppers and grown on an incubated shaker table for two-hours at a 45° angle at 27 °C and 180 rpm to allow the culture to acclimate to the media. After the two-hour incubation period, the headspace was flushed with atmospheric air and resealed, then 500 µL (~ 0.03 atm) of acetylene was introduced into the headspace and the culture was incubated for another two hours at identical conditions. For ethylene analysis, 100 µL of the vial headspace was analyzed via gas chromatography (GC-2014, Shimadzu) with a Flame Ionization Detector using a Restek column 19011 (2 m, 0.75 ID), injection temperature of 80 °C, oven temperature of 40 °C, and FID temperature of 150 °C. A standard curve was prepared with ethylene gas in a similar vial configuration to quantify nitrogenase activity. Protein levels of the whole cells in the vial were quantified using the Biuret assay (Chromý et al. 1974) and a standard curve of Bovine Serum Albumin. All assays were performed in triplicate.

### Ammonium production assays 

To test biological nitrogen fixation by nitrate and urea in *G. diazotrophicus* GABB034 (Δ*amtB1* Δ*amtB2*), we applied the same method previously employed (Dietz et al. 2024) to quantify ammonium accumulation by providing a minimal amount of nitrogen in the form of asparagine (1.25 mM) to enable GABB034 to reach a cell density sufficient to induce aerobic diazotrophic growth. Cell density and ammonium levels were determined daily by measuring optical density at 600 nm and then removing a 1 mL aliquot and centrifuging at 12,000 g to pellet cells and separate the supernatant. The ammonium in the supernatant was quantified using the phthalaldehyde method as previously described (Dietz et al. 2024; Plunkett et al. 2020). All experiments were performed in triplicate.

### Phylogeny tree and enzyme comparisons

16 S rRNA sequences were obtained from published sequences of selected strains. A phylogenetic tree was reconstructed using the Maximum Likelihood method and General Time Reversible model (Nei and Kumar 2000) of nucleotide substitutions and the tree with the highest log likelihood is shown. Branch support was assessed by 1,000 bootstrap replicates (Felsenstein 1985). The initial tree for the heuristic search was selected by choosing the tree with the superior log-likelihood between a Neighbor-Joining (NJ) tree (Saitou and Nei 1986) and a Maximum Parsimony (MP) tree. The NJ tree was generated using a matrix of pairwise distances computed using the General Time Reversible model (Nei and Kumar 2000). The MP tree had the shortest length among 10 MP tree searches, each performed with a randomly generated starting tree. The evolutionary rate differences among sites were modeled using a discrete Gamma distribution across 4 categories (*+ G*, parameter = 0.2999). The analytical procedure encompassed 15 coding nucleotide sequences using 1st, 2nd, 3rd, and non-coding positions with 1,590 positions in the final dataset. Evolutionary analyses were conducted in MEGA12 (Kumar et al. 2024). The final phylogeny tree was visualized and edited using iTOL (Letunic and Bork 2024). Gene presence and absence were determined using available annotated genomic datasets.

## Results

### Azotobacter vinelandii acetylene assays 

**Fig. 1:**
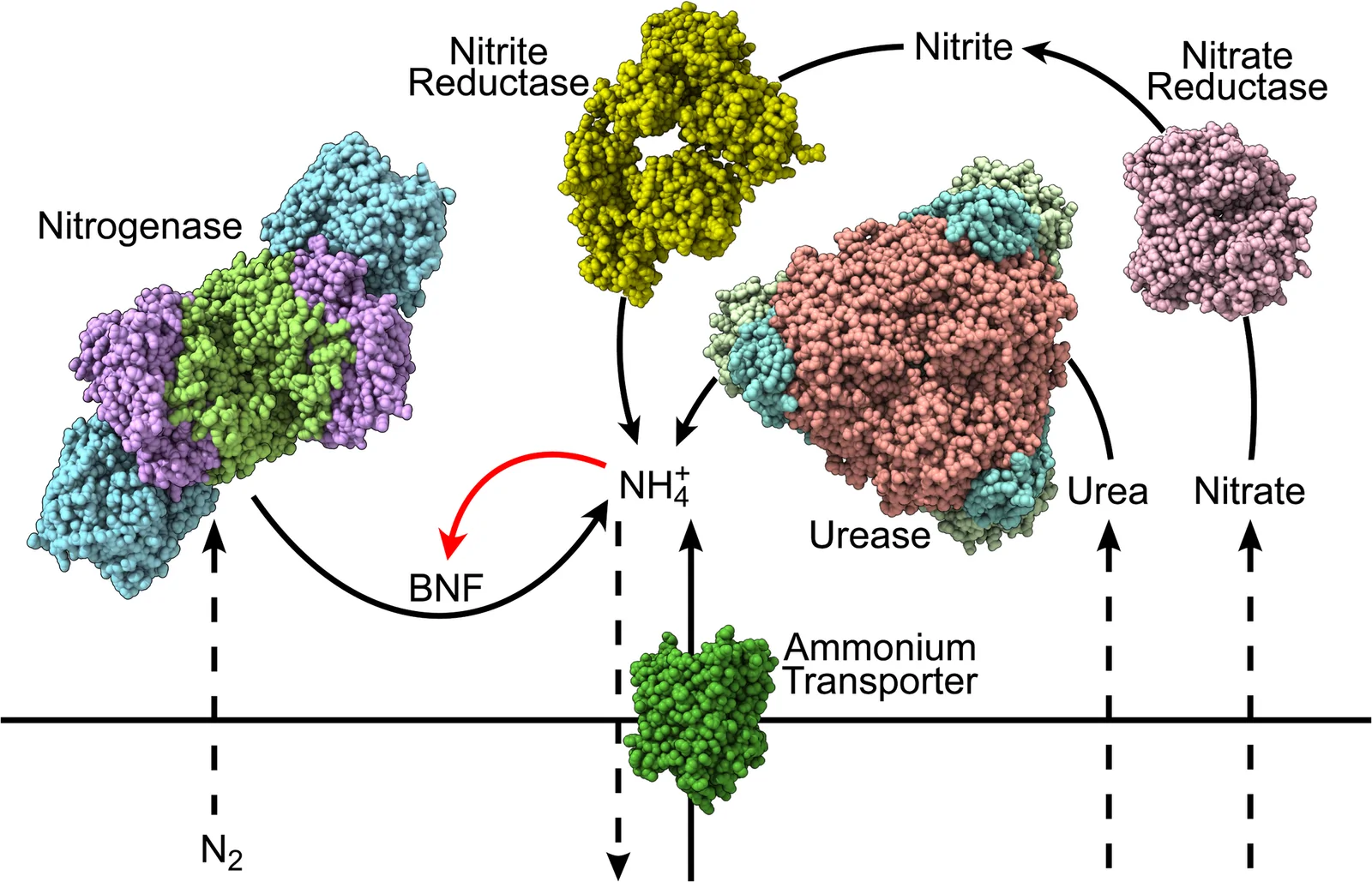
Diagram of nitrogen assimilation pathways in *Azotobacter vinelandii*. Arrows indicate pathways of various nitrogen compounds. The red arrow indicates repression of biological nitrogen fixation (BNF) by ammonium. The nitrogenase structure is based on PDB file 1N2C, urease on 8HCN, the ammonium transporter on 1U7G, nitrate reductase on 2V3V, and nitrite reductase was predicted by AlphaFold from the *A. vinelandii* DJ protein sequence. All molecular structures were generated using ChimeraX

**Fig. 2:**
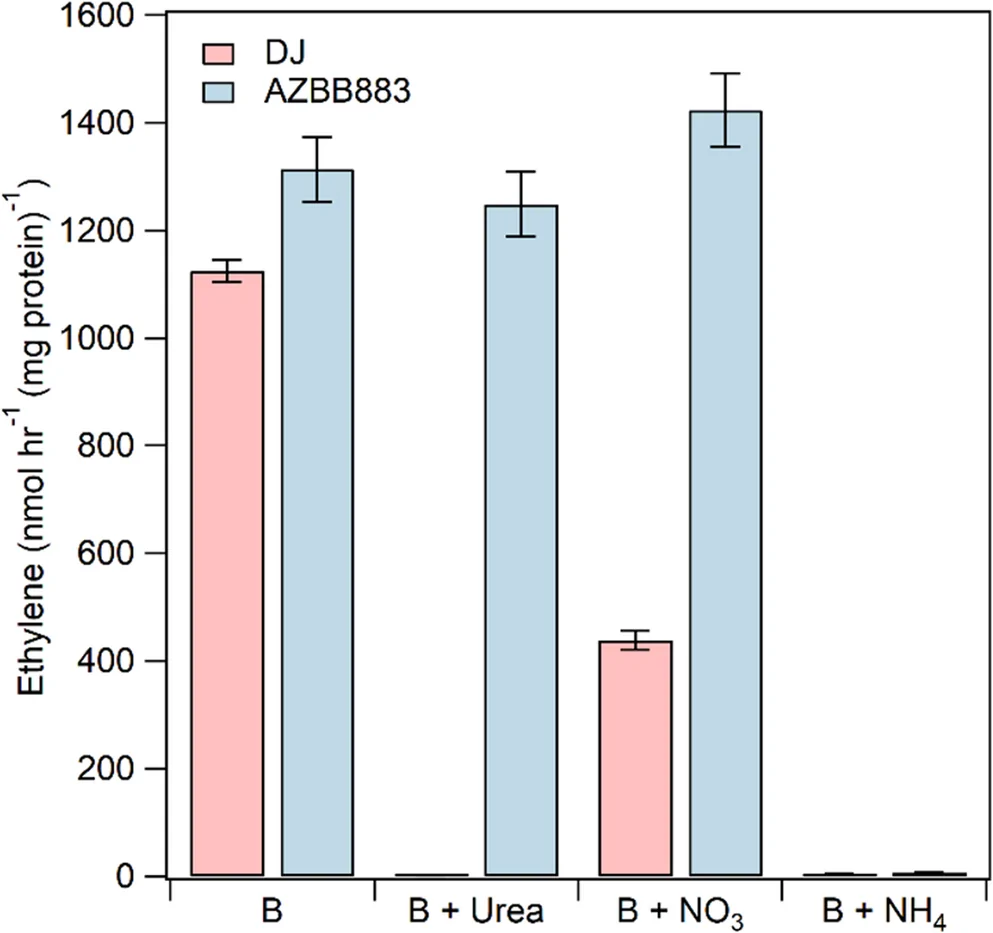
Nitrogenase activity of wild-type *Azotobacter vinelandii* DJ and strain AZBB883 in Burk’s (B) medium and in B medium supplemented with various fixed-nitrogen sources. Shown are nitrogenase acetylene assay results from full cells grown in B medium and in B medium supplemented with 15 mM urea, 15 mM sodium nitrate and 3 mM ammonium chloride. Assays were run for 2 hours in the presence of acetylene. Strain AZBB883 lacks genes for nitrate and nitrite reduction, urea hydrolysis and ammonium transport (Δ*nasAB*, Δ*ureABC* and Δ*amtB*), and was constructed by markerless deletion techniques such that no foreign genes or antibiotic markers are left behind. Results shown are based on triplicate analysis (*n*=3)

Whole cell acetylene assays were employed as a measure of intracellular nitrogenase activity in *A. vinelandii* strains. The wild-type DJ strain of *A. vinelandii* was tested along with the Δ*amtB*, Δ*nasAB* and Δ*ureABC* triple deletion mutant strain AZBB883 (Fig. 1). Both strains exhibited similar intracellular nitrogenase activity levels in the absence of any extraneously provided fixed nitrogen source, although the AZBB883 strain did yield slightly higher levels of ethylene production (~ 16% increase, Fig. 2). The inclusion of urea significantly repressed nitrogenase activity in wild-type in a concentration dependent manner, with 15 mM urea resulting in near total inhibition (> 99%) of nitrogenase activity in *A. vinelandii*, but did not significantly alter the nitrogenase activity in AZBB883 (< 5%). The inclusion of sodium nitrate also impacted nitrogenase activity in a concentration dependent manner in wild type *A. vinelandii*, where 15 mM sodium nitrate exhibited a ~ 61% reduction in nitrogenase activity relative to the control, but again had no significant effect on nitrogenase activity in AZBB883 (~ 8% increase). The inclusion of 3 mM ammonium chloride dramatically repressed nitrogenase activity for both strains (> 99%). These results agree well with our prior studies (Knutson et al. 2018) measuring hydrogen accumulation potential and the concentration dependent inhibition patterns of ammonium versus urea or nitrate. Strain AZBB883 grew diazotrophically with a doubling time of 2.9 h, compared to 3.2 h for the wild-type DJ, which aligns with results obtained in prior studies (Knutson et al. 2018), indicating that there is no growth penalty for the altered AZBB883 under diazotrophic growth. Levels of urea and nitrate selected in these experiments are well above general levels found in agricultural soils (Kabala et al. 2017; Kojima et al. 2006; Miller et al. 2007).

### Gluconacetobacter diazotrophicus ammonium accumulation assays

**Fig. 3:**
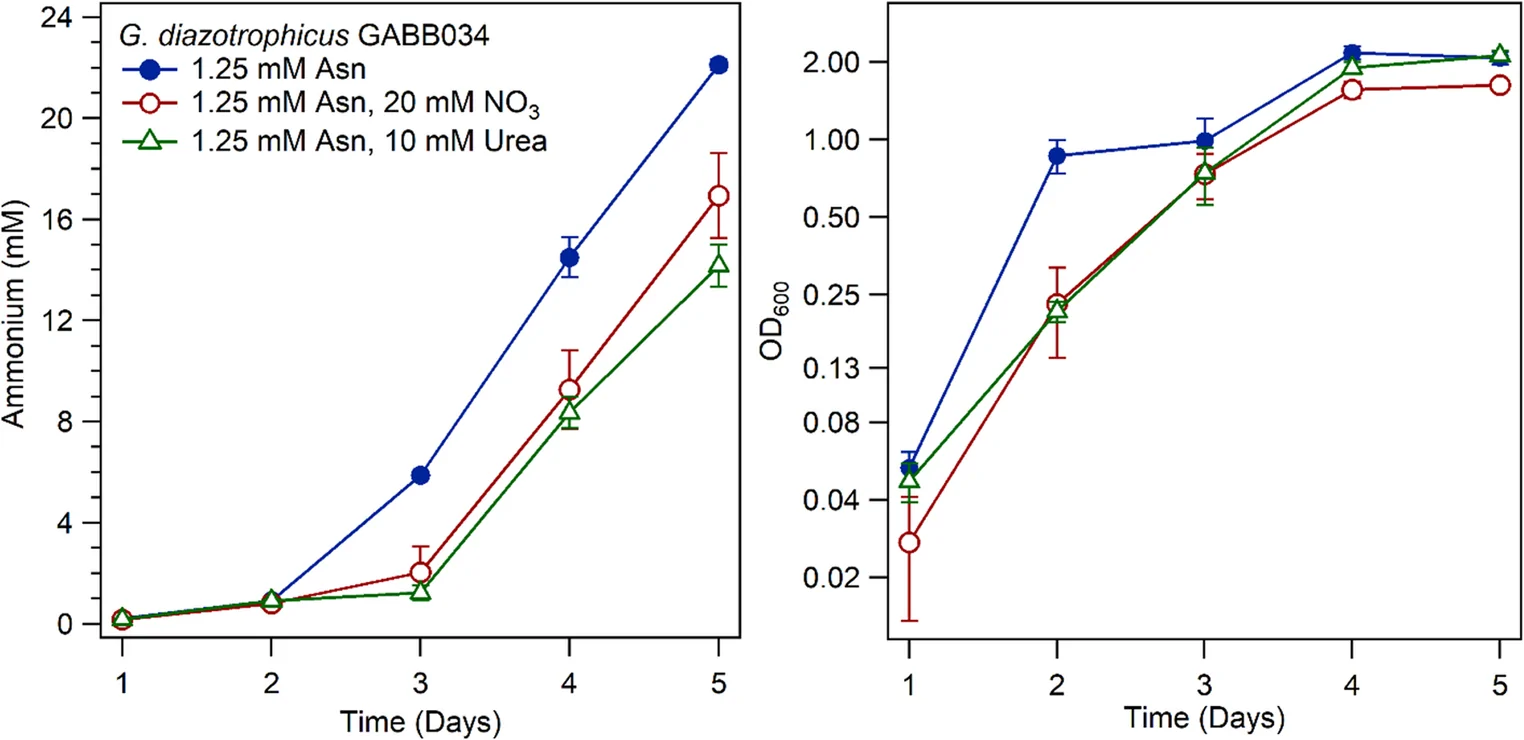
Ammonium accumulation and cell growth in *Gluconacetobacter diazotrophicus* GABB034. Shown above are the ammonium accumulation for the Δ*amtB1* Δ*amtB2 G. diazotrophicus* strain GABB034 grown in ammonium accumulating medium (containing 1.25 mM asparagine) alone and when supplemented with 20 mM sodium nitrate and 10 mM urea. Cultures supplemented with additional nitrogen (nitrate or urea) exhibited a slight lag in initial growth versus the control without further nitrogen. All conditions began to accumulate extracellular ammonium once cells had reached a cell density above 0.5 OD_600_, and rates of ammonium accumulation were similar. Assays were performed in triplicate (*n* = 3)

Unlike *A. vinelandii*, which contains known genes encoding nitrate and nitrite reductases (*nasAB*) and urease (*ureABC*) enzymes, the genome of *G. diazotrophicus* does not apparently contain either of these enzymatic pathways (Bertalan et al. , Giongo et al. 2010). While we were able to successfully perform acetylene assays with *A. vinelandii* strains, *G. diazotrophicus* is not as amenable to whole cell acetylene assays in our hands. As an alternative measure of nitrogenase activity, we previously developed an approach for whole cell ammonium accumulation, using a strain devoid of the genes encoding ammonium transporters AmtB1 and AmtB2 (strain GABB034 (Dietz et al. 2024)). When grown in Burk’s medium supplemented with 35 mM sodium citrate as a buffer and 1.25 mM asparagine to provide sufficient nitrogen to achieve a cell density that enabled aerobic diazotrophic growth, GABB034 began to accumulate ammonium after day 2 (Fig. 3). Once GABB034 had surpassed an OD_600_ of 0.5, it accumulated ammonium at a steady rate for the next 3 days. When provided with urea and sodium nitrate, the growth (as measured by optical density) of GABB034 was delayed versus when these nitrogen sources were not present. Cells achieved an OD_600_ at day 3 that was sufficient to achieve aerobic diazotrophic growth, and the cells began to accumulate extracellular ammonium at a rate that was equivalent to that seen in the absence of the urea and sodium nitrate. These results provide no evidence that urea or nitrate affect nitrogen fixation in *G. diazotrophicus*, and only resulted in a lag in growth before ammonium accumulation began. A similar experiment is difficult to replicate with *A. vinelandii*, because unlike *G. diazotrophicus* strain GABB034, which accumulates mM levels of ammonium following deletion of the *amtB* genes, *A. vinelandii* only releases µM levels of ammonium unless BNF is partially deregulated (Barney et al. 2015; Plunkett et al. 2020).

Lack of known pathways or genes for urease or nitrate reductase does not fully discount the presence of alternative pathways or unknown genes that might function with similar activities. To confirm the lack of any pathways that would funnel nitrogen from urea or nitrate into central metabolism, we substituted the 1.25 mM of asparagine that supports sufficient growth to enter diazotrophic growth for *G. diazotrophicus* (Dietz et al. 2024) with either urea (10 mM) or sodium nitrate (20 mM) as the sole nitrogen source. In the presence of 1.25 mM asparagine, the wild-type strain achieves a density of 0.5 OD_600_ within the course of 48 h (similar to the results for GABB034 in Fig. 3). When we replaced the 1.25 mM asparagine with this excess of nitrogen in the form of either urea or nitrate, the cells of wild-type *G. diazotrophicus* and GABB034 failed to replicate and increase in density, confirming that these substrates are not suitable sources of nitrogen for *G. diazotrophicus*.

### Comparisons of nitrate reductase and urease pathways among diazotrophs 

**Fig. 4:**
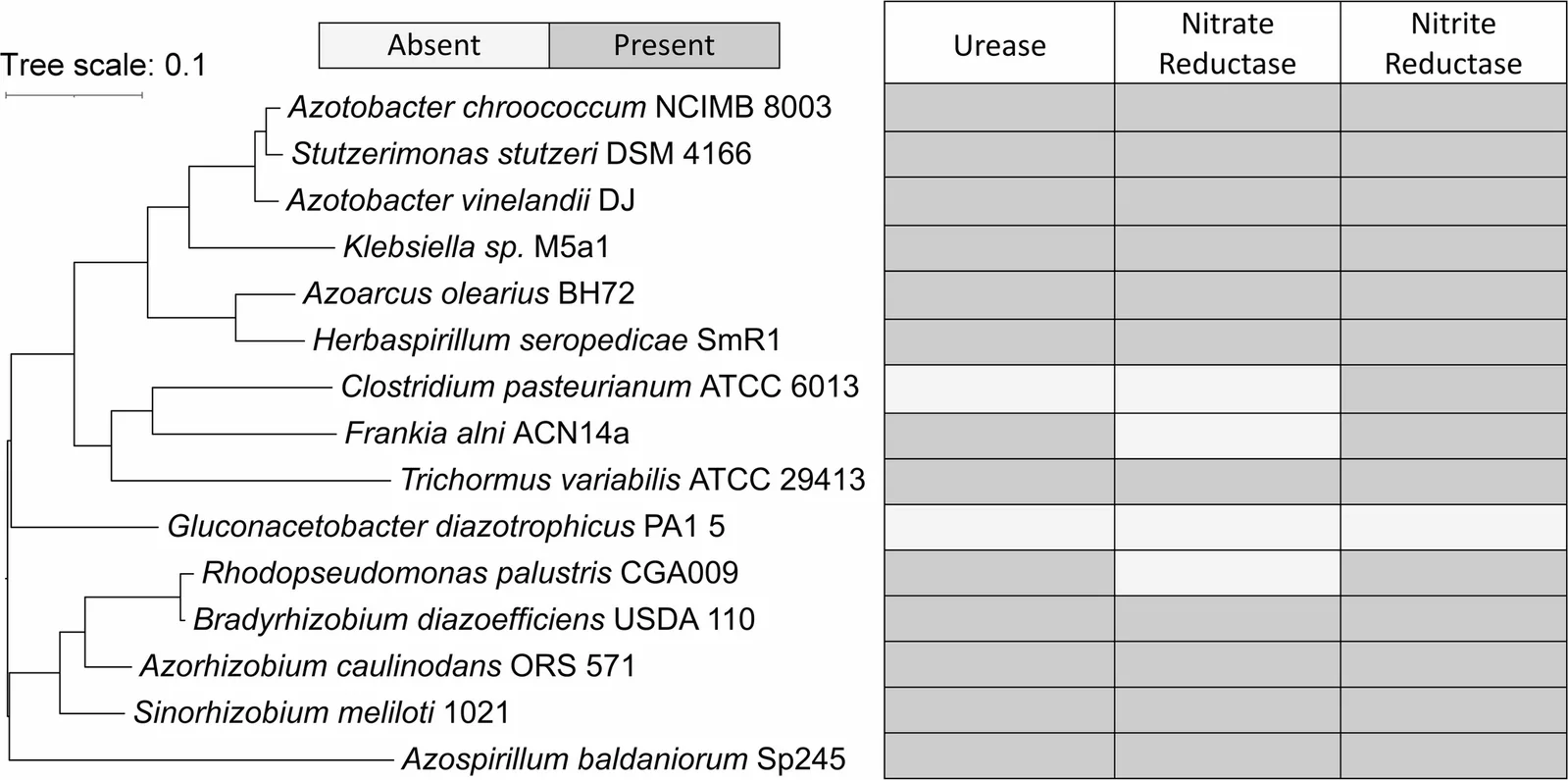
Phylogenetic tree of representative diazotrophic microbes and a table illustrating the presence of pathways converting nitrogen compounds into ammonium

There are many different strains of diazotrophic microbes in consideration for various biofertilizer applications to improve levels of nitrogen provided through BNF. To provide a general picture of how common these pathways are in model diazotrophic species, we selected a variety of strains that contain the genes for BNF and are confirmed to display active nitrogenase activity in the literature. While not exhaustive, this list (Fig. 4) illustrates that most diazotrophs contain pathways to convert urea or nitrate into ammonium when provided in the environment. While several strains lack a homolog for nitrate reductase, only *Clostridium pasteurianum* and *G. diazotrophicus* lack both nitrate reductase and urease homologs in their published genomes.

## Discussion

There have been many efforts taken in recent years to construct nitrogen-fixing (diazotrophic) microbial strains of agricultural significance in an effort to yield enhanced nitrogen production for biofertilizer applications (Bali et al. 1992; Bloch et al. 2020; Shanmugam and Valentine 1975). These biofertilizers have been met with some apprehension in field studies, where they face various obstacles (Franzen et al. 2023; Giller et al. 2025), while others have claimed significant success (Bageshwar et al. 2017; Leaungvutiviroj et al. 2010). Any efforts to encourage adoption of these enhanced microbial strains will likely require the strains to first supplement existing conventional industrial fertilizers that are widely used in agriculture. This requirement for the strains to function within these constraints has the potential to limit any additional nitrogen complementation as biological nitrogen fixation (BNF) is generally tightly regulated and repressed when sufficient plant-available nitrogen is available in the environment (Batista and Dixon 2019; Dixon and Kahn 2004). The choice of industrial fertilizers across the globe varies, with some farmers using urea, while others use ammonium nitrate, anhydrous ammonia, or organic fertilizers derived from animal waste streams (Chataut et al. 2023; Heffer and Prud’homme 2016). The choice of nitrogen fertilizer is influenced by availability, cost and requirements associated with transportation, especially if the percentage of nitrogen is low in the fertilizer source.

*A. vinelandii* is a model strain for the study of BNF, and BNF is tightly regulated in the presence of various available environmental nitrogen sources (Batista and Dixon 2019). While many reports focus on regulation by intracellular metabolites and the specific role that ammonium plays in repressing nitrogenase expression (Batista et al. 2021; Brewin et al. 1999), less attention has been devoted to other forms of fixed nitrogen that are present in or applied to soils (Farzadfar et al. 2021). The inclusion of urea or nitrate in growth medium represses biological nitrogen fixation in wild-type *A. vinelandii*, resulting in diminished nitrogenase activity (Fig. 2). We previously demonstrated that this inhibition in *A. vinelandii* is not specifically related to urea or nitrate, but is instead the result of other enzymatic processes that convert urea into ammonium through the action of urease, or reduce nitrate to ammonium through the action of nitrate and nitrite reductase (Fig. 1 and (Knutson et al. 2018)). For this study, we constructed strain AZBB883 through markerless gene deletions of both urease (*ureABC*) and nitrate/nitrite reductases (*nasAB*) in addition to the ammonium transporter *amtB* (Fig. 1). This strain resulted in a slight increase in nitrogenase activity versus wild-type, likely resulting from the leakage of small quantities of ammonium that results from the removal of the AmtB ammonium transporter (Barney et al. 2015; Zhang et al. 2012), which would require additional nitrogenase activity to compensate for ammonium losses to the extracellular environment. As expected, the removal of these two pathways left AZBB883 insensitive urea or nitrate at concentrations up to 15 mM, resulting in no obvious repression of BNF (Fig. 2). We propose that elimination of these pathways should be considered as a starting point in biofertilizer design strategies since no detrimental diazotrophic fitness loss was observed for this strain.

Our laboratory has also constructed strains of *G. diazotrophicus* that accumulate extracellular ammonium as a result of the loss of the two copies of AmtB that are found in this strain (Dietz et al. 2024). *G. diazotrophicus* has a smaller, streamlined genome in contrast to *A. vinelandii* (Bertalan et al. , Giongo et al. 2010; Setubal et al. 2009), and does not contain any known homologs to urease or nitrate reductase. Based on the absence of these enzymes, we were interested in probing whether biological nitrogen fixation is repressed in the presence of elevated levels of urea and nitrate. Despite various attempts, including efforts to measure activity during active ammonium accumulation, we were not able to perform acetylene activity assays on whole cells of *G. diazotrophicus*. As an alternative, we utilized direct measurement of ammonium accumulation. While our results do indicate that *G. diazotrophicus* is still able to accumulate ammonium in the presence of urea and nitrate, we did find that the presence of these fixed nitrogen sources resulted in a delayed initial cell growth phase. This manifested as a slightly extended lag phase before the cells reached a sufficient cell density to grow diazotrophically under aerobic conditions (Fig. 3). However, once cells reached the required cell density threshold, the rates of ammonium accumulation achieved were equivalent to what was found when urea or nitrate were absent, indicating that these metabolites are not repressing nitrogenase expression or activity in *G. diazotrophicus*. Further tests to see if urea or nitrate could substitute as a nitrogen source in a minimal medium that does support growth in the presence of asparagine confirmed that neither urea or nitrate are able to support the growth of *G. diazotrophicus* in either GABB034 or wild-type cells.

Important to this study, none of the strains described or used in these experiments contain any manipulations aimed to either deregulate or upregulate BNF in *A. vinelandii* or *G. diazotrophicus*. The only modifications that are shared between these two strains is the deletion of the ammonium transporters. *A. vinelandii* contains a single *amtB* gene, and deletion of this gene results in only modest increases in levels of extracellular ammonium (Barney et al. 2015). However, this single *amtB* deletion was sufficient to support a significant amount of secondary biomass production when cultured together with the green alga *Chlorella sorokiniana* (Barney et al. 2015), indicating that the presence of a nitrogen sink likely resulted in an increased flux of ammonium to the non-diazotrophic recipient. This represents an unanticipated driving force that does not directly correlate to the low levels (~ 10 µM) of ammonium that accumulate in the absence of the algal nitrogen sink (Barney et al. 2015). *G. diazotrophicus* contains two copies of the *amtB* gene, and in contrast to *A. vinelandii*, deletion of these two genes (GABB034) results in the accumulation of much higher levels (> 10 mM) of ammonium to the extracellular space, reaching levels that can only be achieved in *A. vinelandii* when BNF is at least partially deregulated (Barney et al. 2015; Dietz et al. 2024). By deleting the urease and nitrate/nitrite reductases from *A. vinelandii*, we created a strain (AZBB883) that is insensitive to nitrate or urea even when high levels (15 mM) are present, well above levels that are typical of agricultural soils (Pinton et al. 2016). Because ammonium is a central metabolite involved in many biological processes, and also the direct result of the nitrogenase enzyme (Barney et al. 2006), it is doubtful that ammonium inhibition can ever be fully removed without altering specific enzymes involved in BNF regulation. Indeed, ammonium levels of 3 mM were sufficient to completely arrest nitrogenase activity in *A. vinelandii* for the wild-type and AZBB883 strains (Fig. 2). Increases in ammonium accumulating in the extracellular space with GABB034 did not slow overall nitrogenase activity in this strain (near linear rate of accumulation for all conditions from days 3–5 in Fig. 3). Urea and nitrate, however, can be viewed as terminal products or nitrogen sinks if these strains lack the ability to hydrolyze or reduce these substrates. Neither of the strains studied here contain any foreign genes, such that they are not transgenic strains. Since they have only lost genes, and since the entire genes were removed, it will be difficult for these strains to regain these enzyme activities as a result of any single spontaneous mutation or further gene disruptions, leading to improved long-term stability of the strains versus modifications that upregulate BNF.

Our laboratory recently demonstrated that urea production in *A. vinelandii* can be dramatically enhanced from prior levels by first converting urea into a terminal product, and by then deregulating arginine production and adding a functional arginase (Barney and Dietz 2025). In that construct, as was done here, there were no manipulations made to BNF regulation, and the elevated urea levels were simply the result of a terminal sink, which did not result in an obvious growth penalty, as assessed by doubling time during diazotrophic growth versus the wild-type DJ strain (Barney and Dietz 2025).

One benefit of these strains is that it should be possible to incorporate these into current agricultural practices that use non-ammonium based fertilizers (NaNO_3_, KNO_3_ or urea) while not repressing BNF. Additionally, starting with a strain that lacks urease and nitrate/nitrite reductase would ensure that these could be incorporated into a broader range of agricultural applications without the concern that they will compete for either of these plant-available nitrogen substrates. As a further assessment of the potential for application of this strategy, we surveyed a variety of model diazotrophs studied for their agricultural significance to assess how broadly urease and nitrate/nitrite reductase are distributed among these strains (Fig. 4). We selected only model strains that are highly studied and have completed genomes that are supported by experimental evidence of BNF. While not an exhaustive list, this does represent a variety of strains, and as can be seen in Fig. 4, the lack of urease or nitrate/nitrite reductases is not common, with *G. diazotrophicus* standing out as an outlier among this list. Regulation of BNF in various diazotrophs is highly variable (Batista and Dixon 2019), with some strains using a *nifLA* regulator system such as is found in *A. vinelandii*, while others do not contain the *nifL* repressor, similar to *G. diazotrophicus*. However, regulation of BNF is not solely attributed to *nifA*, and can be further regulated by PII proteins or NtrB-NtrC regulator systems. For this reason, testing the effects of various nitrogen sources on BNF in different bacteria will likely need to be assessed for each target biofertilizer strain. While beyond the scope of this current study, it will be interesting in the future to investigate the potential to apply this strategy to other biofertilizer strains.

Since the intention behind many laboratory-modified diazotrophic biofertilizer strains is to provide additional nitrogen to the plant, microbial engineers must not only consider how these strains perform within the laboratory, but also consider what will be required of these strains when introduced back into the microbial communities and natural plant microbiomes where they will be integrated. Most biofertilizer strains are anticipated to supplement current industrial fertilizers, and it is important that strains are able to enhance nitrogen in the presence of other nitrogen inputs. Strains will also need to compete with native soil and plant microbiomes, and contend with external nitrogen inputs or sinks, which may limit any potential immediate advantage they would have over non-diazotrophs when growing in a complex community. Additionally, plant breeders, agronomists and horticulturists will need to consider methods of nitrogen application and selection of optimal conditions to ensure that engineered biofertilizers deliver the benefits of their designs. By constructing or selecting strains devoid of urease and nitrate reductase pathways, we ensure here that *G. diazotrophicus* and *A. vinelandii* will not compete for urea or nitrate forms of nitrogen, and are primed to provide additional nitrogen in the presence of urea and nitrate fertilizers.

## Data Availability

All data generated or analyzed during this study are included in this published article or are available from the corresponding author on reasonable request.

## Declarations

This article does not contain any studies with human participants or animals performed by any of the authors.
